# Angiotensin Converting Enzyme (ACE): A Marker for Personalized Feedback on Dieting

**DOI:** 10.3390/nu12030660

**Published:** 2020-02-28

**Authors:** Shilpa Tejpal, Narinder Sanghera, Vijayalaxmi Manoharan, Joan Planas-Iglesias, Claire C Bastie, Judith Klein-Seetharaman

**Affiliations:** 1 Systems Biology and Biomedicine, Division of Biomedical Sciences, Warwick Medical School, University of Warwick, Gibbet Hill, Coventry CV4 7AL, UK; tejpalshilpa@gmail.com (S.T.); narinder.sanghera@googlemail.com (N.S.); vijimanoharan@gmail.com (V.M.); jo.planas@gmail.com (J.P.-I.); C.C.Bastie@warwick.ac.uk (C.C.B.); 2 Institute for Digital Healthcare, Warwick Manufacturing Group, University of Warwick, Coventry CV4 7A, UK

**Keywords:** obesity, ACE, weight loss, biomarkers, personalized feedback, dieting

## Abstract

Angiotensin Converting Enzyme (ACE) expression and activity is associated with obesity. ACE is a circulating factor that predicts sustained weight loss over a time frame of months. Here, we evaluate whether ACE might also be an early marker (over a 24-hour period) for weight loss. 32 participants (78% females; BMI 28.47 ± 4.87kg/m2) followed a 1200KCal diet with an optional daily (<250KCal) snack and were asked to use an in-house generated health platform to provide recordings of food intake, physical activity and urine collection time and volume. Following a day of dieting, ACE levels in urine negatively correlated with weight loss (*p* = 0.015). This reduction in ACE levels was significantly more robust in individuals with a BMI > 25 (*p* = 0.0025). This study demonstrated that ACE levels correlate with BMI and weight loss as early as after 1 day of dieting, and thus ACE could be a potential early “biofeedback” marker for weight loss and diet efficiency.

## 1. Introduction

Obesity is a rapidly growing health problem affecting over one-third of the world’s population [1,2]. It is associated with a number of chronic diseases such as dyslipidemia, hypertension, type 2 diabetes, stroke and several cancers [3,4]. Genetic, behavioral, socio-economic and environmental factors all contribute to obesity [5] but the condition is also treatable to a great extent [6]. There is a range of approaches available to reduce weight using surgical, drug-based interventions or by following different diet programs [7,8,9]. The first line of treatment is dietary management along with behavior management and physical activity [10]. 

To date, no dietary intervention combines molecular measurements with digital technology tracking life-style parameters such as food intake or exercise, despite the availability of many food tracker apps and websites [11]. All weight loss programs give a broad description of the diet plan, which is often arbitrary. While most diets are conducted on a long-term (e.g.; several weeks, months or years), limited work is focused on a short-term modulation of biomarkers in response to a diet. In addition, every individual has a different metabolism and responds differently to a diet plan [11]. Thus, personalized optimization of a diet plan needs to be implemented to determine how diet parameters can be adapted to an individual’s metabolism. Therefore, there is an unmet need to identify markers that could be correlated with lifestyle and dietary habits. 

Recording of eating patterns through questionnaires with or without computer assistance has been recognized as an effective step in managing obesity [12,13,14]. However, regardless of interface, reporting of too low or too high caloric/energy intake as compared to true intake is a well-documented problem [11,15,16]. This unconscious bias is associated with under-realization and in some scenarios even over-estimation of foods eaten [11,17]. Even if food tracking was entirely accurate, it might not provide sufficient motivation for people to lose weight [11,18]. Thus, an approach to weight loss is needed that is more directly linked to the desired outcome, weight loss, itself [11,19]. A quantitative and science-based biomarker of weight loss is needed to provide immediate/short-term biological feedback loop during dieting.

Urinary metabolic markers for cardiovascular disease, blood pressure and adiposity have been identified [20,21]. Several metabolomics studies involving untargeted proton (1H) nuclear magnetic resonance spectroscopy (NMR) and ion exchange chromatography (IEC) with human and mice urine samples have identified metabolites associated with BMI and adiposity [20,22]. Quantitative biomarkers have not been considered for providing early (especially over a 24-hour period) feedback to an individual undergoing a weight loss intervention. 

The extent of reduction in ACE has been shown through blood profiling for protein and steroid hormones to be an important predictor for sustained weight loss on long time scales [23]. The participants who kept their weight off after one year also had decreased ACE concentrations at the end of an eight-week low caloric diet. ACE is a zinc metallopeptidase involved in the conversion of Angiotensin (Ang) I to Angiotensin II [23,24,25,26]. Ang I is obtained by cleavage of Angiotensinogen (AGT) with the help of renin. Ang II is a well-known for its role in increased blood pressure and retention of salt and water [24,27,28]. In addition, animal models have shown that increased adipose specific angiotensin (AGT) expression and secretion is involved in adipose tissue development [29,30].

The requirements for dieting feedback are more immediate than general adiposity markers, or the long-term ACE weight loss marker. We have previously established that skipping at least one meal (breakfast, lunch or dinner) results in weight loss regardless of which meal was skipped [11]. Despite meticulously keeping records of food intake, individuals still found losing weight challenging [31,32,33,34,35]. Our goal is to identify a molecular marker that associated with weight loss so that it could be used for feedback. This feedback could potentially be given as a “traffic-light signals”, indicating the effect of daily actions (e.g. caloric intake) on their long-term goal, weight loss. The scope of this study is to identify ACE as a potential marker for weight loss over 24-hour periods. We have previously identified insulin and lactate as potential early markers for dieting [11], and here we are presenting data supporting ACE as an additional potential marker.

## 2. Materials and Methods 

### 2.1. Study Design 

**Figure 1:**
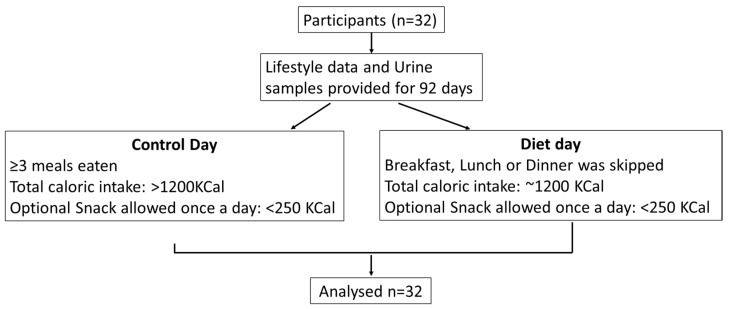
Study design.

The study was approved by the Warwick Medical School Ethics committee BSREC (protocol identification REGO-2014-1318). The design and dietary intervention have been described in detail previously [11]. All participants provided informed written consent and were 18 years or older and not taking any medication. It was an observational study where the individuals used an in-house developed health platform, the Digital Health Platform (DHP), available at personalhealth.warwick.ac.uk, google play and app stores to enter food diaries, urine volume and physical activity. The urine volume provided for each sample was used to calculate the amount of ACE in each sample. The only main constraint for a diet day was the omission of one of the three regular meals with total caloric intake of 1200 KCal. The participants were allowed an optional daily snack of <250Kcal (Figure 1). Caloric intake parameters were retrieved based on meal information provided by the user and converted into KCal by multiplying with 4, 4 and 9 per gram of carbohydrates, protein and fat, respectively, as described [11]. 

Participants were asked to collect data for at least one 24-hour period. Each 24-hour period strictly included all data for the entire period including first and last urine sample collection and weight measurements. The number of 24-hour periods during which each participant collected data was chosen freely by the participants because each period was considered as a separate unit. The average number of 24-hour periods was 3 days/participant, and the maximum number of data was entered by one participant who provided 21 periods. The individuals were asked to collect samples for at least one diet day and one control day (non-dieting). If participants collected samples for more than one 24-hour period, they could do so for as many diet days as they chose. After a day of intervention, to understand how the variables vary on the next morning, the participants were asked to collect a sample the day after a day of an intervention (i.e. the 24-hour period begins with collection of the first sample on the morning of the day of the intervention and ends on the following morning with collection of the last sample). Therefore, we have a following day sample. The participants weighed themselves every morning before breakfast. They were instructed to maintain a routine that would make weighing as reproducible as possible. The individuals collected urine samples every time they emptied the bladder over the given 24-hour period, and so the total number of samples collected over a 24-hour period may vary. However, they all contain at least one morning and one following morning sample. 

### 2.2. Measurement of Angiotensin Converting Enzyme

ACE was measured (based on manufacturer’s instructions) using a Human ACE DuoSet ELISA (DY 929) from R&D Systems, UK. ACE levels were measured in each urine sample collected over a day. The data analysis is done based on different variables given in the Table 1.

### 2.3. Measurement of Insulin and Lactate

Urine samples were collected and registered in the DHP and their insulin and lactate content was quantified using a Mesoscale Discovery Human Insulin Kit and a fluorescence based assay, respectively, as described previously [11,36].

### 2.4. Statistical Analysis

Statistical analyses were performed using IBM SPSS Statistics 24 and R. Bivariate Pearson Coefficient analyses was used to calculate the association between different variables. The Benjamini–Hochberg procedure was used for controlling the false discovery rate [37]. 

## 3. Results

### 3.1. Study Characteristics

The observational study was publicized through flyers, newsletter and website advertisement at the University of Warwick and through word of mouth. Participants were recruited over a period of 6 months. A total of 32 participants were recruited (Figure 1). The individuals also provided food intake information and urine samples for 92 days. Out of these 92 days, 22 were control days and 70 were diet days. The participants used the DHP to maintain food diaries, physical activity, urine collection time and volume. 78% of the participants were females and 22% were males. The mean BMI of the cohort was 28.47 ± 4.87 kg/m2 (mean ± standard deviation). There was a total of 12 participants with BMI ≤ 25 and 20 individuals with BMI > 25. The participants followed a meal skipping plan, as described in Methods section. The values were then used to define a total of 14 parameters relating to biomarker profile or lifestyle data entered (Table 1). 

### 3.2. ACE Levels Correlate with Life-Style Data

**Figure 2:**
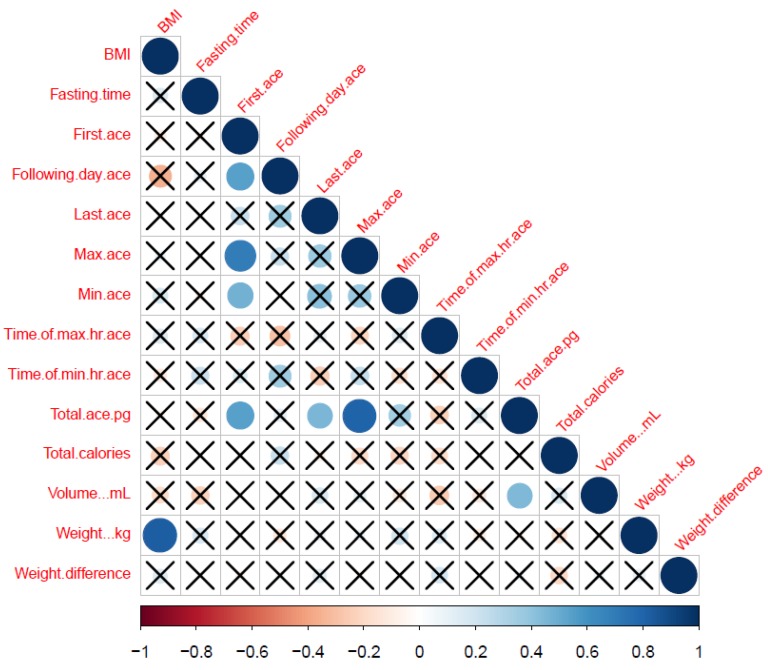
Correlation Matrix for ACE derived and other relevant dieting parameters. Correlations between the variables (shown in Table 1). They were scaled from 1.0 to −1.0. Blue indicates positive correlation while red indicates negative correlation. X indicates no correlation between the two parameters which is significant at *p* = 0.01. The size of the circles corresponds to the strength of the correlation. The bigger the circle, the stronger is the correlation between 2 variables.

The cross-correlation matrix of all the 14 extracted variables from biomarker profiles and the DHP has been shown (for the complete cohort) in Figure 2 for an overall summary. Most of the participants provided data for 2–3 days. First ACE correlated with following day, maximum, minimum and total ACE (Figure 1), but not with time of maximum and minimum ACE in 24-hour, last ACE, total calories, urine volume, weight and weight difference. On applying Benjamini–Hochberg procedure for multiple correction, correlation between following day ACE and BMI; last ACE and first ACE, following day ACE, maximum ACE and minimum ACE; and minimum ACE and total ACE was lost.

### 3.3. Effects of BMI on ACE Levels

**Figure 3:**
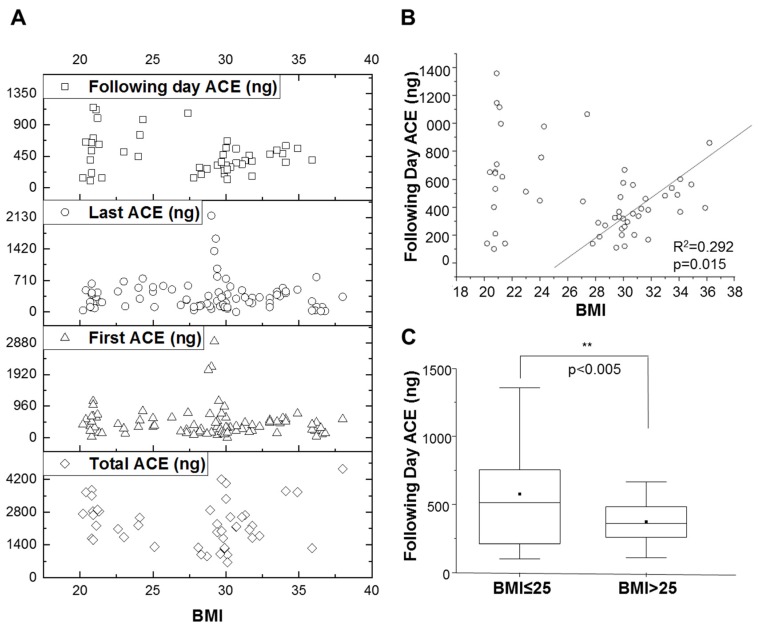
Effects of BMI on ace levels. (A)Spread of ACE values in comparison to BMI; (B) Trend in following day ACE and BMI. BMI ≥ 25 group and following day ACE was positively correlated (R^2^ = 0.292) significant at *p* = 0.015; (C) ACE response is dependent upon BMI. Following day ACE correlation was studied in different BMI groups. Significance level marked as **p < 0.01.

We analyzed the association between ACE and pre-defined BMI groups and correlations between ACE levels at different times (e.g.: first collection, last collection) in these BMI groups. There was an association between BMI and ACE levels on the following day (Figure 3A). Moreover, a linear trend was observed in BMI > 25 group for the following day ACE levels with R^2^ = 0.292 and significant at *p* = 0.015 (Figure 3A,B). The decrease in ACE levels after a day of dieting was higher in individuals with BMI ≥ 25 (*p* = 0.0025; Figure 3C).

### 3.4. Dependence of Weight Loss on ACE Level

Encouraged by the correlation between BMI and following day ACE, next we investigated if there is also a correlation between ACE levels and the desired parameter, “weight loss”. We did not find a correlation between weight loss and ACE levels when examining the entire cohort (Figure 1). However, a strong association between the following day ACE levels and weight loss was observed in individuals with a BMI ≥ 25, significant at R^2^ = 0.274 and *p* = 0.015 (Figure 3). 

### 3.5. Correlation among ACE, Insulin and Lactate

**Figure 4:**
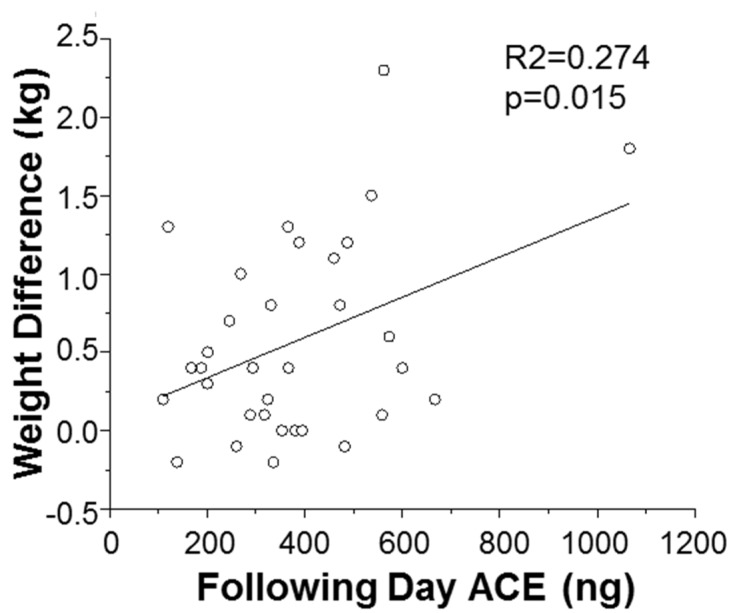
ACE as an early biomarker for weight loss in individuals with a BMI ≥ 25. Plot of following day ACE against weight difference. R^2^ = 0274 and *p* = 0.015.

Previously, we have studied lactate and insulin concentrations in the samples provided by the participants [11]. We therefore conducted Pearson Correlation to determine if there was an association between ACE, lactate and insulin variables. First ACE correlated positively with first insulin values, whilst last ACE correlated negatively with maximum and total insulin values (Figure 4). Following day ACE also had a positive correlation with following day, maximum, minimum and total insulin values (Figure 4). On applying Benjamini–Hochberg procedure for multiple comparisons, correlations between first ACE and first insulin; following day ACE and following day insulin and maximum insulin; last ACE and maximum insulin and total insulin was lost. 

**Figure 5:**
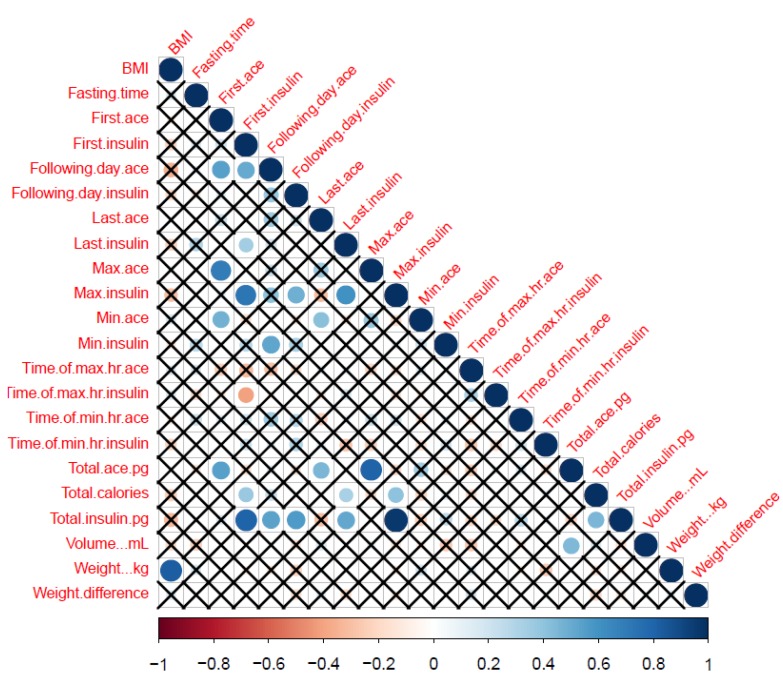
Correlation Matrix for ACE and Insulin. Correlations between the parameters were scaled from +1.0 to −1.0. Blue indicates positive correlation while red indicates negative correlation. X indicates no correlation between the two parameters which is significant at *p* = 0.05.

**Figure 6:**
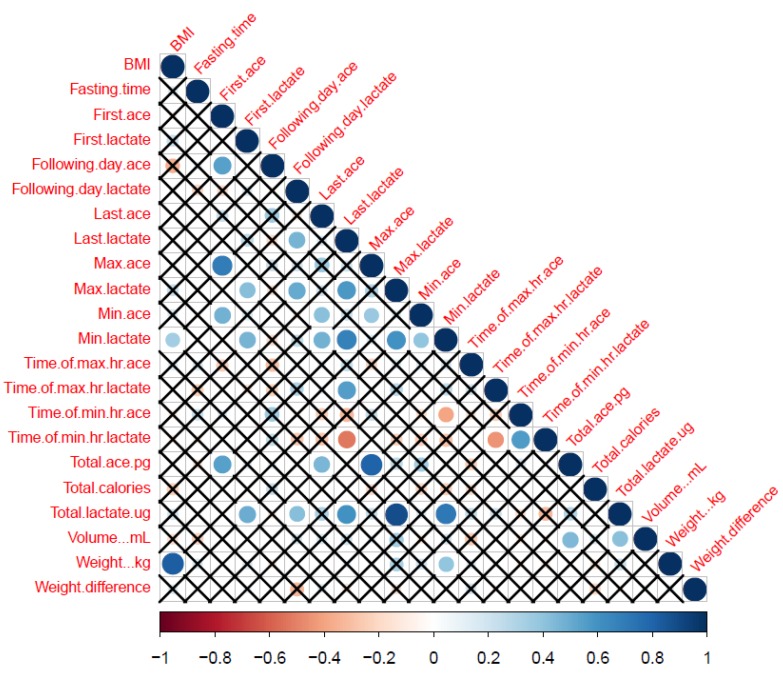
Correlation Matrix for ACE and Lactate. Correlations between the parameters were scaled from +1.0 to −1.0. Blue indicates positive correlation while red indicates negative correlation. X indicates no correlation between the two parameters which is significant at *p* = 0.05.

To a lesser extent, ACE values also correlated with lactate values. Last ACE correlated positively with maximum and total lactate (Figure 5). Maximum ACE correlated with maximum lactate and minimum ACE correlated to minimum lactate values (Figure 5). We have found previously that insulin and lactate levels (especially following day levels) correlated with weight loss. Correlation with following day ACE variable showed that it might be usable in an exchangeable manner while analyzing weight loss in an individual. On applying Benjamini–Hochberg procedure for multiple correction, correlation between last ACE and total lactate and maximum ACE and maximum lactate was lost (Figure 6).

## 4. Discussion

ACE has been identified as an important predictor for sustained weight loss through profiling for blood protein and steroid hormones after a low caloric diet for 8 weeks [23]. Therefore, ACE appeared to be a marker correlated with weight loss on a long term. However, our goal was to identify markers on a very short term, as early as 24 hours after the start of a diet regimen, to provide feedback to the individuals and potentially reassure the individuals about the efficiency of their diet (if it is helping in losing weight). Thus, it was investigated whether correlation could be found between urine ACE levels and parameters such as BMI and weight loss.

Following day ACE levels correlated with BMI however, a stronger association was observed in those individuals where it matters the most, with BMI ≥ 25 (overweight and obese group). Thus, ACE might be a good “indictor” on diet efficiency in such individuals where it could be used as a feedback on weight management success. Our current study provides us with the insight needed to design a future study appropriately. In particular, we intend to conduct a study with larger numbers of participants (over a longer periods of time) and where participants will be randomly assigned to intervention group with or without personalized molecular feedback.

The main aim of the study was to identify if there might be any correlation between molecular data and weight loss. This was an observational study consisting of the relatively small number of 32 mostly female individuals, making small size a major limitation in the generality of the conclusions that can be drawn from our study. The study was advertised and conducted at the University of Warwick. The participants were mostly students or staff members at the University of Warwick. The sample size of the current study is small, and the observations cannot be extrapolated to the whole population. A larger cohort study of more diverse participants needs to be conducted to confirm the trends observed here. Also, the treatments (which meal to skip and on what day) were not assigned randomly. The participants were given the liberty to choose what days to diet, as well as what meals to skip. Different lifestyles of everyone might also generate a sampling bias as they were not chosen at random. 

While our study indicates that skipping a meal is beneficial in losing weight, literature also shows that skipping meals, especially breakfast, can be associated with increased risk of obesity, hypertension, diabetes, cardiovascular disease and metabolic syndrome [38]. In the United States, in a cohort study of 6550 adults of which 5.1% never consumed breakfast, 10.9% rarely consumed breakfast, 25% consumed breakfast some days, and 59% consumed breakfast every day, those individuals who never consumed breakfast had a higher risk of stroke-related mortality with hazard ratio (HR) of 3.39 [39]. Another large-scale study consisting of 6764 adults indicated that a 1% increase in total energy intake at breakfast was associated with a lower weight gain [40]. Skipping breakfast has been associated with a 21% higher risk of developing type 2 diabetes [41]. A meta-analysis of fasting studies showed that individuals who fasted on a regular basis had an odd’s ratio (OR) of 0.65 for coronary artery disease compared with individuals who did not fast routinely [41]. Eating frequency has also been associated with obesity and its associated conditions. A cross-sectional study with 499 individuals found that people who ate ≥ 4 times in a day had lower risk of obesity when compared to people who ate ≤ 3 times in a day [42]. Another study found higher eating frequency to be associated with lower concentrations of LDL cholesterol [41]. 

Circulating levels of ACE are influenced by the genetic polymorphism [23,43] known to be related to obesity [30,44]. The association between ACE and obesity has been suggested to be the result of the role of angiotensin II in the development of adipose tissue [29,45,46]. Angiotensinogen and angiotensin II are present in human adipose tissue [45,47,48,49]. Even though in our study only healthy individuals participated, it is important to realize that individuals taking medication can have altered protein levels. For example, when gauging albuminuria reduction with lotrel in diabetic patients (GUARD study) with hypertension, it was found that a combination of ACE inhibitor and calcium channel blocker led to higher decrease in urinary albumin excretion in comparison to a β-blocker and a diuretic combination for type 2 diabetics [50]. Along with this, a decrease in the glomerular filtration rate was observed [50]. A combination therapy with ACE inhibitor and angiotensin receptor blocker lead to higher decrease in urinary protein levels in comparison to when used on its own [51]. 

We have previously established that decreased last and following day insulin and lactate levels correlate with weight loss [11]. Association between following day ACE and last insulin has been established in this study. The correlation between different ACE and insulin and lactate variables show that these values could be used interchangeably to provide molecular feedback. Thus, a person on a diet, in the future, could measure their ACE values and decide if it is acceptable to eat another meal that day, or what type of meal it should be. We also have identified lemon extract as a mean to regulate ACE levels in urine. From the cellular studies, it has been identified that lemon could potentially acts as an ACE inhibitor leading to improved insulin sensitivity [28]. In the previous study, we have previously shown that more than 30% individuals were interested in knowing their metabolic profile. This showed that finding molecular markers apart from daily weighing could be helpful for some people, a hypothesis that requires testing in future studies. 

There are a number of potential sources of errors in this study. First, participants were asked to record their data in the DHP. They might have forgotten or neglected to enter data, leading to a response bias. Misreporting of food diaries is known to be a limiting factor in observational studies [52]. Misreporting involves both, over- and under-reporting of food eaten (too little or too much food, respectively, compared to true food intake) affecting the conclusions from the data collected [53]. Because users measured their own weight, improper measurement might generate response bias. Similarly, users were instructed to store urine samples immediately at 4 or −20 °C, but if they did not follow these instructions and samples were kept at higher temperatures for extended periods of time, this would be expected to affect sample stability. This in turn would lead to inaccurate measurement of biomolecules. Other daily activities such as physical activity also might have affected a participant’s weight. Although the DHP allowed entries for physical activity, very few entries were made, making it another potentially confounding variable.

The length and cost of the assay for ACE, and the need for urine samples were also limitations. The assay for ACE required a laboratory setting, making it not yet feasible to conduct a long-term study or investigate the effect on behavior in situ. We are currently in the process of developing rapid, cheap and home-based sensors for weight loss biomarkers in urine [36], which would enable us to address these limitations in the future. These sources for potential bias could have affected our study and our findings need to be further corroborated and validated in future efforts.

## 5. Conclusion

In conclusion, this study investigated a molecular feedback approach to assist dieting efforts. ACE values showed correlations with BMI and weight difference, when analyzing population with BMI ≥ 25. In particular, the study demonstrated that ACE levels vary within a 24-hour interval after following a calorie-restricted diet. In conclusion, the inter-individual variation of ACE has shown that it could potentially be used as an early biofeedback marker on dieting and weight loss for individuals that are overweight or with obesity.

## Figures and Tables

**Table 1:** Variables found. Variables extracted from ACE profile obtained from the urine samples and entries on the digital health platform, DHP (https://personalhealth.warwick.ac.uk/).

Variable	Description
Extracted from Biomarker Profile	
Volume of urine in 24 hour (mL)	Total volume of urine produced over a 24-hour period
First ACE value on diet day	Obtained from urine sample just before the first meal of the day
Last ACE value on diet day	Obtained from the last urine sample of the diet day
Total ACE value of 24 hour	Summation of ACE values obtained from all the urine samples provided over a 24-hour period
Following day ACE	Obtained from the first urine sample at the end of the 24-hour period on the following day
Minimum ACE value in 24-hour	The smallest amount of ACE amongst all the urine samples provided over a 24-hour period
Maximum ACE value in 24-hour	The largest amount of ACE amongst all the urine samples provided over a 24-hour period
Time of maximum ACE in 24-hour	Time stamp of the urine sample entry in the DHP that corresponds to the maximum ACE value
Time of minimum ACE in 24-hour	Time stamp of the urine sample entry in the DHP that corresponds to the minimum ACE value
**Variable from Digital Health Platform (DHP) entries**	
Fasting time	Obtained by subtracting the time of the second meal from the first meal of the day
Total calories	Obtained from summation of the caloric information of the food entries on DHP over a 24-hour period
Weight	Entered by the participant (measured every day in the morning)
Weight difference	Weight before breakfast minus weight following morning at the same time
BMI	Either entered by the participant or calculated based on the weight and height entry of the participant
